# Trait disinhibition mediates the associations of depressive symptoms and BMI in a non-clinical cohort of lean and individuals with obesity

**DOI:** 10.3389/fpsyg.2026.1705171

**Published:** 2026-04-02

**Authors:** Imke Schamarek, Anna Bautz, Michael Stumvoll, Matthias Blüher, Peter Kovacs, Anke Tönjes, Kerstin Rohde-Zimmermann

**Affiliations:** 1 Medical Department III - Division of Endocrinology, Nephrology and Rheumatology, University of Leipzig Medical Center, Leipzig, Germany; 2 Helmholtz Institute for Metabolic, Obesity and Vascular Research (HI-MAG); Helmholtz Center Munich at the University of Leipzig and the University Clinic Leipzig, Leipzig, Germany; 3 Institute for Diabetes and Obesity, Helmholtz Zentrum München, Leipzig, Germany

**Keywords:** depressive symptoms, disinhibition, mediation, obesity, trait eating behavior

## Abstract

**Background:**

Depressive symptoms affect eating behavior and body mass index (BMI). We investigated their relationship in a non-clinical cohort without depressive or eating disorders and explored whether trait eating behavior mediates the association between depressive symptoms and BMI.

**Methods:**

Seventy-seven participants (62.3% women, aged 20–69 years) from the Obese Taste Bud study, with varying weight status and no depressive or eating disorders, completed the General Depression Scale (GDS), Three-Factor Eating Questionnaire (TFEQ), and Food Craving Questionnaire. Multiple linear regression analyses were conducted to examine the associations of depressive symptoms with trait eating behavior and BMI. Mediation analyses were performed using Hayes’ PROCESS macro in the Statistical Package for Social Sciences (SPSS).

**Results:**

Higher depressive symptom scores predicted greater trait disinhibition (*β* = 0.228, *p* = 0.044), emotional eating (*β* = 0.307, *p* = 0.009), and food craving (*β* = 0.309, *p* = 0.009) but not trait hunger, restraint eating, or cognitive restraint (all *p* < 0.05). Furthermore, higher depressive symptom scores predicted higher BMI (*β* = 0.399, *p* < 0.001). Greater trait disinhibition (*β* = 0.377, *p* < 0.001) and food craving (*β* = 0.335, *p* = 0.002) were associated with a higher BMI. Trait disinhibition partially mediated the relationship between depressive symptoms and BMI (indirect effect: B = 0.0948, standard error [SE] = 0.0575; 95% confidence interval [CI]: 0.0022, 0.2250).

**Conclusion:**

In non-clinical cohorts, depressive symptoms that do not meet the criteria for depressive disorders meaningfully influence stable patterns of unfavorable eating behaviors and body weight. Trait disinhibition was identified as a mediator linking depressive symptoms and BMI, highlighting a potential behavioral mechanism through which depressive symptoms may contribute to obesity.

## 1 Introduction

The effects of depressive symptoms on trait eating behavior—defined as enduring individual patterns in responsiveness to food-related cues—are well documented (Paans et al., 2018; Konttinen et al., 2010; Torales et al., 2025; Anil et al., 2025). Accordingly, depressive symptoms are associated with increased trait food craving, which is the tendency to experience frequent and intense desires for specific food types, typically high in sugar, fat, or carbohydrates (Muftuoglu et al., 2018; Graybeal et al., 2024). In addition, positive associations of depressive symptoms and trait emotional eating (tendency to eat in response to negative emotions), trait hunger (susceptibility to internal hunger cues to trigger eating), and trait disinhibition (tendency to eat in response to emotional, situational, or environmental cues, rather than physiological hunger) have been described. However, reported associations with trait restraint eating (controlling food intake to prevent weight gain or promote weight loss) are inconsistent (Paans et al., 2018; Konttinen et al., 2010; Bégin et al., 2012; Mutwalli et al., 2025). These associations may partly stem from shared neural pathways for emotional and eating behavior, and it appears that eating behavior directed toward highly palatable foods might contribute to counter-regulation of negative emotions (Hepworth et al., 2010; Coccurello, 2019).

Both unfavorable trait eating behaviors and depression have been linked to higher BMI and an increased risk of obesity (de Wit et al., 2010; Luppino et al., 2010; Löffler et al., 2015; Jung et al., 2025; Shell et al., 2024; Putra et al., 2025). Although a bidirectional association between depression and obesity is acknowledged, and longitudinal studies strongly suggest a direct effect of depression on the development of obesity, the underlying mechanisms remain to be elucidated (de Wit et al., 2010; Luppino et al., 2010).

Much of the existing research has focused on clinical populations comprising patients with diagnosed mood disorders and/or eating disorders or obese individuals seeking treatment (Bégin et al., 2012; Luppino et al., 2010; Werrij et al., 2006). However, studies on how depressive symptoms relate to trait eating behavior and BMI in non-clinical cohorts remain limited, where similar patterns may still emerge but go unrecognized. Addressing these interrelations in non-clinical settings provides a deeper understanding of the early risk mechanisms that precede unfavorable eating behavior and its consequences for weight regulation.

This study aims to investigate the association between depressive symptoms, trait eating behavior, and BMI in a population without diagnosed mood or eating disorders. We hypothesize that depressive mood increases unfavorable trait eating behaviors and is associated with a higher BMI. We further explore the mediating role of trait eating behavior to clarify the relationship between depressive symptoms and BMI, with the goal of establishing prevention and intervention strategies in non-clinical populations.

## 2 Methods

### 2.1 Recruitment and study population

Participants were recruited as part of the Obese Taste Bud Study between 2020 and 2024 (NCT04633109 or DRKS00022950) through advertisements on the institutional homepage and through flyers distributed across the campus of the University Clinic of Leipzig and the Helmholtz Institute for Metabolic, Obesity and Vascular Research (HI-MAG), Helmholtz Center Munich at the University of Leipzig, and the University Clinic Leipzig. Potential participants were screened for eligibility during a telephone interview, in which a trained research assistant assessed the inclusion and exclusion criteria: Male and female participants were required to be between 18 and 69 years of age. Participants suffering from severe kidney, heart, liver, neurological, or mental diseases were excluded, as were those diagnosed with a malignant disease or those who had undergone radiation, chemotherapy, or recent surgery. For the present analysis, participants were further excluded if diagnosed with an eating disorder, a depressive disorder, or taking antidepressant medication. Furthermore, the analysis was limited to participants with complete datasets for all relevant variables, resulting in a cross-sectional analysis of 77 participants.

### 2.2 Study design

The study design is described in greater detail elsewhere (Kersten et al., 2024). Briefly, participants arrived at the outpatient unit of the University Clinic of Leipzig or HI-MAG at 7:30 a.m. after at least 12 h of overnight fasting. Upon arrival, all participants provided written informed consent, including permission for their anonymized data to be used in future analyses. The present re-analysis was conducted under the original ethical approval and is consistent with the conditions outlined in the consent form. All procedures met the standards of the Declaration of Helsinki and were approved by the ethics committee of the Medical Faculty of the University of Leipzig, Leipzig, Germany (protocol code 011/20-ek; date of approval 19.03.2020). Participants’ characteristics and their medical and behavioral data were assessed during a standardized interview and through self-report questionnaires. Among others, the following information was collected: Current dieting was assessed via self-report as a dichotomous variable (yes/no). The degree of present hunger was quantified using a visual analog scale anchored with 0 (not hungry) and 100 (very hungry). In addition, the following validated questionnaires were applied:

#### 2.2.1 General Depression Scale

Depressive symptoms were assessed using the General Depression Scale (GDS), the German adaptation of the Center for Epidemiological Studies Depression Scale, which is particularly suitable for screening depressive symptoms in non-clinical settings (Radloff, 1977). The GDS is a self-reporting questionnaire designed to measure the frequency and severity of depressive symptoms in the general population over the past week. It consists of 20 items, covering affective, cognitive, and somatic aspects of depression, each rated on a 4-point Likert scale ranging from 0 (rarely or never) to 3 (majority of the time or all of the time), resulting in a total score ranging from 0 to 60. Higher scores indicate greater depressive symptoms. The GDS has been widely validated for use in the general population, demonstrating strong internal consistency (Cronbach’s alpha: 0.85–0.92) and good construct validity (Radloff, 1977).

#### 2.2.2 Three-Factor Eating Questionnaire

The German version of the 51-item Three-Factor Eating Questionnaire (TFEQ) was used to assess characteristics of the participants’ eating behavior (Schober, 1991; Stunkard and Messick, 1985). All items are coded with 0 or 1. The scoring followed the same procedure as proposed in the original instrument, yielding the subscales cognitive restraint, disinhibition, and hunger. We also included the subscales based on the factor structure identified in a large validation study of the German version of the TFEQ: Uncontrolled eating contains 11 items from the original hunger and disinhibition factors; emotional eating contains three items of the original disinhibition factor and assesses eating caused by emotional triggers such as feeling anxious or depressed; cognitive restraint contains 15 restraint items of the original TFEQ (Löffler et al., 2015). It has demonstrated good internal consistency (Cronbach’s alpha: 0.78–0.84) and good construct validity in non-clinical populations (Löffler et al., 2015).

#### 2.2.3 Food Craving Questionnaire—Trait-reduced

To assess the general tendency to experience food craving, the Food Craving Questionnaire—Trait-reduced (FCQ-T-r) was used (Meule et al., 2014). The FCQ-T-r is a 15-item self-report instrument designed to measure the frequency and intensity of food craving as a stable trait. Items are rated on a 5-point Likert scale from 1 (never) to 5 (always), with higher scores indicating stronger trait-like food cravings. It has demonstrated excellent internal consistency (Cronbach’s alpha: 0.90) and good construct validity in non-clinical populations (Meule et al., 2014).

### 2.3 Anthropometric measures

Anthropometric measurements were performed by a trained research assistant with prior experience in standardized anthropometric assessment. Before study initiation, the assessor completed protocol-specific training to ensure strict adherence to standardized measurement procedures in accordance with established guidelines. Body weight (kg) was measured using a calibrated digital scale, and height (cm) was measured using a wall-mounted stadiometer (Seca, Hamburg, Germany). Participants were assessed wearing light clothing and without shoes. All equipment was calibrated regularly in accordance with the manufacturer’s recommendations. Body mass index (BMI) was calculated as body weight (kg) divided by body height squared (m^2^).

### 2.4 Statistical analysis

Data were analyzed using SPSS version 27 (IBM, Ehningen, Germany) and GraphPad Prism Version 10 (GraphPad Software, Inc., California, USA). An *a priori* power calculation was not performed, as the study represents a retrospective analysis of data collected for a different primary objective. Therefore, the sample size was determined by the available dataset rather than by power considerations. Descriptive data were reported as median and interquartile range. The Mann–Whitney U test was used to assess group differences between men and women for continuous data, whereas the chi-square test was applied for categorical data. Spearman’s rank correlation coefficient was applied to analyze associations between depressive symptoms, BMI, trait eating behavior scores, and other participants’ characteristics as potential confounding variables. The False Discovery Rate (FDR) was used to adjust for multiple comparisons. A multiple linear regression analysis was conducted to test whether depressive symptoms (GDS) predict relevant outcome measures, controlling for potential confounders. A mediation analysis was conducted using model 4 of the PROCESS macro (version 4.2; Hayes, 2022) in SPSS (Hayes, 2022). This model tests the indirect effect of an independent variable (X; depressive symptoms; GDS) on a dependent variable (Y; BMI) through a mediator (M; trait eating behavior), while also allowing the inclusion of covariates previously identified in correlation analyses. Based on previous correlation analyses, mediation analyses controlled for age and current dieting, which were entered as covariates in the model and included as predictors of both the mediator and the outcome variable. The indirect effect was evaluated using a non-parametric bootstrapping procedure with 5,000 resamples, and bias-corrected 95% confidence intervals (CI) were generated. An indirect effect was considered statistically significant if the CI did not include zero. Assumptions of linear regressions (multicollinearity, normality of residuals, and homoscedasticity) were checked and met. An alpha level of <0.05 was considered statistically significant.

## 3 Results

### 3.1 Participants’ characteristics

Participants in this study were predominantly female (62.3%) and aged 20–69 years. BMI ranged from 18.64 to 29.71 kg/m^2^; the majority were of normal weight, without significant differences between men and women. Current depressive symptoms ranged from 0 to 31 and did not differ between men and women. Men and women differed significantly in body height and weight, with men, on average, being taller and heavier. Furthermore, women scored significantly higher on the TFEQ-emotional eating subscale. As men and women did not show significant differences in depressive symptoms, BMI, or any trait eating behavior, except for emotional eating, the following analyses were not further stratified by sex but were adjusted for potential confounding by sex where appropriate. Participants’ characteristics are summarized in Table 1.

**Table 1:** Participants’ characteristics stratified by sex.

Participants’ characteristics	Sample (*N* = 77)	Men (*N* = 29; 37.7%)	Women (*N* = 48; 62.3%)	*p*-value
Body weight (kg)	75.30 (22.95)	78.40 (19.85)	69.75 (24.77)	**0.015**
Height (cm)	172 (13.65)	177.30 (9.25)	167.00 (10.75)	**< 0.001**
BMI (kg/m^2^)	23.87 (8.08)	23.88 (9.23)	23.65 (8.93)	0.462
Weight status				0.436
Normal-weight, *N* (%)	44 (57.1)	18 (62.1)	26 (54.2)	
Overweight, *N* (%)	19 (24.7)	7 (24.1)	12 (25.0)	
Obesity, *N* (%)	14 (18.2)	4 (13.8)	10 (20.8)	
Age (years)	32.00 (21.5)	30.00 (21.50)	32.00 (22.00)	0.658
Smoking *N* = yes/no (%)	9/68 (11.7/88.3)	3/26 (10.3/89.7)	6/42 (12.5/87.5)	0.541
Current hunger (mm)	40.0 (40.00)	50.00 (32.50)	35.0 (40.00)	0.385
Depressive symptoms	9.00 (10.00)	9.00 (7.00)	8.0 (12.75)	0.632
Current dieting, *N* = yes/no (%)	10/67 (13.0/87.0)	3/26 (10.3/89.7)	7/47 (14.6/85.4)	0.435
Trait eating behavior				
Trait food craving	29.0 (18.50)	29.00 (15.50)	31.00 (19.75)	0.344
Trait uncontrolled eating	4.00 (4.00)	4.00 (4.00)	4.00 (3.00)	0.544
Trait restraint eating	5.00 (5.00)	4.00 (4.50)	6.00 (6.00)	0.544
Trait cognitive restraint	7.00 (6.00)	6.00 (6.00)	7.50 (7.75)	0.627
Trait emotional eating	0.00 (2.00)	0.00 (1.00)	1.00 (2.00)	**0.046**
Trait hunger	4.00 (4.00)	4.00 (4.00)	4.00 (4.00)	0.661
Trait disinhibition	6.00 (5.50)	6.00 (6.00)	6.00 (5.50)	0.804

Data are presented as median (interquartile range). The Mann–Whitney U test and the chi-square test were used to assess group differences between men and women. A *p*-value of <0.05 was considered statistically significant (bold). Smoking was defined as smoking at least one cigarette per day, each day of the week. Current hunger was assessed using a 100-mm visual analog scale (0 = not hungry to 100 mm = very hungry). Depressive symptoms were assessed with the General Depression Scale. Self-reported current dieting was assessed with a dichotomous item (yes/no). Trait eating behavior was assessed with the Three-Factor Eating Questionnaire. Trait food craving was assessed with the Food Craving Questionnaire—Trait- reduced. BMI, body mass index; *N*, number of participants.

### 3.2 Correlation analyses of depressive symptoms, trait eating behavior, and BMI

Table 2 presents Spearman’s rank correlation coefficients for the associations between depressive symptoms, trait eating behavior, and BMI, as well as potential confounding variables. Depressive symptoms positively associated with trait food craving, emotional eating, and disinhibition after adjusting for multiple testing, while a significant association with trait hunger did not remain significant. Trait restraint eating, cognitive restraint, and uncontrolled eating were not associated with depressive symptoms. Depressive symptoms were further positively associated with BMI. Additionally, trait disinhibition and food craving were positively associated with BMI, with the associations remaining significant after adjusting for multiple comparisons. Positive associations of trait hunger, emotional eating, and uncontrolled eating with BMI did not remain significant after adjusting for multiple comparisons, and neither trait restraint eating nor cognitive restraint was associated with BMI. Further analyses focus only on associations between depressive symptoms, trait eating behavior, and BMI that remained significant after adjusting for multiple comparisons (indicated in bold in Table 2).

**Table 2:** Association of depressive symptoms, BMI, trait eating behavior, and potential covariates.

*N* = 77	BMI	Age	Sex	Current dieting	Current hunger	Smoking	Depressive symptoms
Depressive symptoms	0.331**	−0.057	0.055	−0.252*	0.225	0.143	-
Trait food craving	0.339**	−0.078	−0.109	−0.183	0.077	0.157	0.335**
Trait disinhibition	0.425***	0.011	−0.028	−0.330**	−0.001	0.088	0.314**
Trait hunger	0.271*	0.025	0.050	−0.080	0.135	−0.027	0.222*
Trait restraint eating	0.170	0.008	−0.070	−0.247*	0.073	−0.014	0.054
Trait cognitive restraint	0.153	0.023	−0.056	−0.255*	0.041	0.014	0.057
Trait emotional eating	0.237*	−0.057	0.229*	−0.277*	0.170	0.044	0.336**
Trait uncontrolled eating	0.284*	−0.029	−0.002	−0.174	−0.066	−0.037	0.180
BMI	**-**	0.259*	0.068,	−0.299,	0.122	0.134	-

Spearman’s rank correlation coefficient was used to assess associations in 77 participants. A *p*-value of <0.05 was considered statistically significant. The results are presented as correlation coefficients (*r*), with *p* < 0.05*, *p* < 0.01**, *p* < 0.001***. The False Discovery Rate method was applied to adjust for multiple testing, and associations that remained significant are highlighted in bold. Smoking was defined as smoking at least one cigarette per day, each day of the week. Current hunger was assessed using a 100-mm visual analog scale (0 = not hungry to 100 mm = very hungry). Depressive symptoms were assessed using the General Depression Scale. Trait eating behavior was assessed with the Three-Factor Eating Questionnaire. Trait food craving was assessed with the Food Craving Questionnaire—Trait-reduced. Self-reported current dieting was assessed using a dichotomous item (yes/no). BMI, body mass index; *N*, number of participants.

### 3.3 Linear regression analyses

Depressive symptoms significantly predicted trait disinhibition, food craving, and emotional eating, controlling for potential confounding variables identified in previous correlation analyses. Covariate selection adhered to a rigorous, predefined statistical workflow aimed at comprehensive control of potential confounding. Variables were considered covariates if they had shown associations in prior correlation analyses and were retained irrespective of the loss of statistical significance after correction for multiple comparisons. Accordingly, BMI and current dieting were included as covariates in regression models in which depressive symptoms served as the predictor and trait disinhibition and trait food craving constituted the outcome variables. Additionally, sex was entered as a covariate in regression models specifying depressive symptoms as the predictor and trait emotional eating as the outcome. The regression coefficients from the multiple linear regression analyses are summarized in Table 3. Depressive symptoms, trait disinhibition, and trait food craving significantly predicted BMI, controlling for potential confounding variables including age and current dieting, as identified in previous correlation analyses. The regression coefficients from the multiple linear regression analyses are summarized in Table 4.

**Table 3:** Multiple regression analyses predicting trait eating behavior.

*N* = 77	Outcome								
	Trait disinhibition			Trait food craving			Trait emotional eating		
Predictor	*β*	SE	*p*	*β*	SE	*p*	*β*	SE	*p*
Depressive symptoms	0.228	0.54	**0.044**	0.309	0.227	**0.009**	0.307	0.020	**0.009**

Standardized regression coefficients of multiple regression analyses, including depressive symptoms as a predictor and trait eating behavior as an outcome, controlling for BMI and current dieting as potential confounding variables in *N* = 77 participants. In the analyses with trait emotional eating as an outcome, sex was additionally included as a potential confounding variable. A *p*-value of < 0.05 was considered statistically significant (bold). Depressive symptoms were assessed with the General Depression Scale. Trait disinhibition and emotional eating were assessed with the Three-Factor Eating Questionnaire. Trait food craving was assessed with the Food Craving Questionnaire—Trait-reduced. *β*, standardized regression coefficient; SE, standard error.

**Table 4:** Multiple regression analyses predicting BMI.

*N* = 77	Outcome BMI		
Predictor	*β*	SE	*p*
Depressive symptoms	0.399	0.115	**<0.001**
Trait disinhibition	0.377	0.250	**<0.001**
Trait food craving	0.335	0.335	**0.002**

Standardized regression coefficients of multiple regression analyses including depressive symptoms and trait eating behavior as predictors and BMI as the outcome, controlling for age and current dieting as potential confounding variables, in *N* = 77 participants. A *p*-value of <0.05 was considered statistically significant (bold). Depressive symptoms were assessed with the General Depression Scale. Trait disinhibition was assessed with the Three-Factor Eating Questionnaire. Trait food craving was assessed with the Food Craving Questionnaire—Trait-reduced. *β*, standardized regression coefficient; SE, standard error.

### 3.4 Mediation analyses

**Figure 1:**
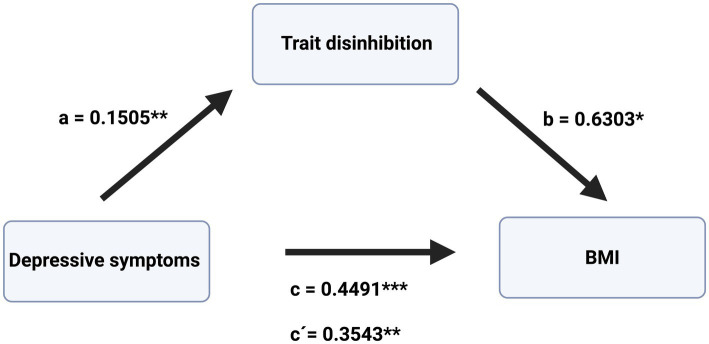
Mediation model including depressive symptoms as an independent variable, trait disinhibition as a mediator, and BMI as a dependent variable. Results of the mediation model, including depressive symptoms as an independent variable, trait disinhibition as a mediator, and BMI as a dependent variable, indicate partial mediation. *c*, total effect; *c*′, direct effect; *a* and *b*, regression coefficient of paths *a* and *b*, respectively. *p < 0.05, **p < 0.01, ***p < 0.001. Created with BioRender.

**Figure 2:**
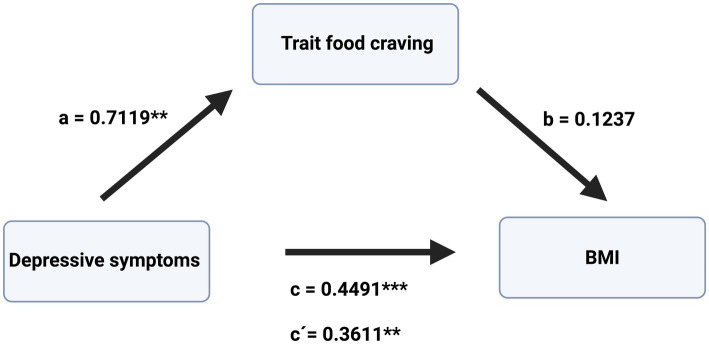
Mediation model including depressive symptoms as an independent variable, trait food craving as a mediator, and BMI as a dependent variable. Results of the mediation model, including depressive symptoms as an independent variable, trait food craving as a mediator, and BMI as a dependent variable. Trait food craving did not mediate the depressive symptom-BMI link. *c*, total effect; *c*′, direct effect; *a* and *b*, regression coefficient of paths *a* and *b*, respectively. *p < 0.05, **p < 0.01, ***p < 0.00.1. Created with BioRender.

Following the results of the correlation and regression analyses, mediation analyses were conducted using the PROCESS macro (model 4) in SPSS (Hayes, 2022). In a first model, trait disinhibition was entered as the mediator (M), depressive symptoms as the independent variable (X), and BMI (Y) as the dependent variable. In a second model, trait food craving was entered as a mediator (M), with depressive symptoms as the independent variable (X) and BMI as the dependent variable (Y). Age and current dieting were included as covariates, as these variables had been identified as potential confounders in preceding correlation analyses (Table 2). To maintain a rigorous statistical workflow, these covariates were retained in the model despite losing statistical significance following adjustment for multiple testing. The results are summarized in Table 5 and further illustrated in Figures 1, 2, showing disinhibition and food craving as mediators, respectively. The results indicate significant effects of depressive symptoms on disinhibition (path a) and of disinhibition on BMI (path b), controlling for age and current dieting. Furthermore, the total effect of depressive symptoms on BMI was significant (path c), and after accounting for trait disinhibition as mediator, the direct effect of depressive symptoms on BMI was also found to be significant (path c′). The indirect effect of depressive symptoms on BMI through trait disinhibition (path a*b) was also significant, as indicated by a 95% bias-corrected bootstrapped confidence interval of [0.0022, 0.2250] based on 5,000 bootstrap samples. Since both the direct and indirect effects were significant, the results indicate partial mediation of trait disinhibition in the association between depressive symptoms and BMI. The overall model explained a significant proportion of variance in BMI, R^2^ = 0.3484, *F*(5.0, 72.0) = 9.6253, *p* < 0.001.

**Table 5:** Mediation analyses of trait eating behavior linking depressive symptoms with BMI.

*N* = 77	*B*	SE	*p*-value	Bootstrap 95% CI	
Trait disinhibition					
Path a (X → M)	0.1505	0.0569	0.0101	0.0370	0.2640
Path b (M → Y)	0.6303	0.2854	0.0304	0.0613	1.1993
Path c (Total effect)	0.4491	0.1098	0.0001	0.2303	0.6680
Path c′ (Direct effect)	0.3543	0.1150	0.0029	0.1250	0.5835
Path a*b (Indirect effect)	0.0948	0.0575		0.0022	0.2250
Trait food craving					
Path a (X → M)	0.7119	0.2544	0.0066	0.2048	1.2189
Path b (M → Y)	0.1237	0.858	0.1538	−0.0474	0.2948
Path c (Total effect)	0.4491	0.1098	0.0001	0.2303	0.6680
Path c′ (Direct effect)	0.3611	0.1303	0.0071	0.1013	0.6208
Path a*b (Indirect effect)	0.0881	0.0681		−0.0053	0.2546

Mediation models were conducted with depressive symptoms (General Depression Scale) as the independent variable (X), BMI as the dependent variable (Y), and trait disinhibition (model 1) or trait food craving (model 2) as mediators (M), controlling for age and current dieting in both models. Path a represents the effect of depressive symptoms on the mediator (trait disinhibition or trait food craving). Path b represents the effect of the mediator on BMI. Path c describes the total effect of depressive symptoms on BMI. Path c′ represents the direct effect of depressive symptoms on BMI after accounting for the mediator, and the indirect effect represents the effect of depressive symptoms on BMI through the mediator (trait disinhibition or trait food craving). BMI, body mass index; X, independent variable (depressive symptoms); M, mediator (trait disinhibition/trait food craving); Y, dependent variable (BMI); B, effect size; SE, standard error, CI, confidence interval.

Furthermore, the effect of depressive symptoms on trait food craving (path a) was found to be significant, whereas the effect of trait food craving on BMI (path b) was not significant (Table 5). After accounting for trait food craving as a mediator, the direct effect (path c′) of depressive symptoms on BMI remained significant. However, the indirect effect of depressive symptoms on BMI through trait food craving (path a*b) was not significant, as indicated by a 95% bias-corrected bootstrapped confidence interval of [−0.0077, 0.2531] based on 5,000 bootstrap samples. The results indicate that trait food craving did not mediate the relationship between depressive symptoms and BMI. The overall model predicting BMI was statistically significant, R^2^ = 0.2910; *F*(3.00;73,00) = 9.4211, *p* < 0.0001, indicating that the predictor variables—depressive symptoms, trait food craving, and covariates—accounted for a substantial proportion of variance in BMI; however, this effect was not mediated by trait food craving.

## 4 Discussion

The results indicate an association between depressive symptoms and unfavorable trait eating behavior and higher BMI, even when the criteria for depressive disorders are not met. This study is the first to demonstrate that trait disinhibition partially mediates the relationship between depressive symptoms and BMI, highlighting the relevance of these interrelations in non-clinical populations.

### 4.1 Association of depressive symptoms with trait eating behavior and BMI

Our study shows that higher depressive symptom scores predicted increased trait disinhibition, food craving, emotional eating, and BMI. Consistent with these results, previous studies in non-clinical populations have reported similar depressive symptom scores, also without significant gender differences (Konttinen et al., 2010; Wang et al., 2022). In addition, consistent with our results, higher depressive symptom scores predicted greater trait disinhibition, and both factors were positively associated with BMI in young Asian adults (Wang et al., 2022). Depressive symptoms were also positively associated with trait emotional eating and uncontrolled eating in this population (Wang et al., 2022). Furthermore, greater trait disinhibition and trait hunger have also been reported in overweight and obese women with higher depressive symptoms (Bégin et al., 2012). Across women in various BMI categories, emotional eating was positively associated with depressive symptoms and BMI (Clum et al., 2014). In the present analyses, the positive association between emotional eating and BMI did not remain significant after correction for multiple comparisons. Similarly, emotional eating was positively associated with depressive symptoms but not BMI in Mexican students (Lazarevich et al., 2023). A population-based study confirmed a positive association between trait emotional eating and depressive symptoms and reported that both depressive symptoms and emotional eating were positively correlated with BMI (Konttinen et al., 2010). However, these studies did not exclude, or did not report excluding, individuals with diagnosed mood or eating disorders or those taking antidepressant drugs, which may impact generalizability. A European cohort of obese adults, excluding those with current depressive or eating disorders or those under antidepressant treatment, found depressive symptoms positively associated with trait emotional and uncontrolled eating, contrasting the present findings (Paans et al., 2018). Variations may arise from different assessment tools and the inclusion of individuals with prior depression, with potential effects on current behavior. A study in women without mental disorders positively linked depressive symptoms with trait uncontrolled eating, while not associating them with trait emotional eating, partially supporting the present results (Fernandez et al., 2024). Research on the association between depressive symptoms and restrained eating is inconsistent, possibly reflecting the complexity of restrained eating addressed below (Paans et al., 2018; Bégin et al., 2012; Werrij et al., 2006). However, the non-significant findings observed in the present analyses, particularly after adjusting for multiple comparisons, which stand in contrast to the well-documented associations between depressive symptoms and trait emotional, uncontrolled, and restrained eating behavior, are likely attributable to limited statistical power. *Post hoc* power calculations conducted with G*Power, using effect sizes reported in the literature (*r* ≈ 0.20–0.30), indicated that a sample of approximately *N* = 84–193 would have been required to achieve 80% power at *α* = 0.05 (Faul et al., 2007). Thus, the study may have been slightly underpowered to detect moderate associations and clearly underpowered to detect small effects, with further reduction of statistical power after correction for multiple comparisons. Therefore, null findings should be interpreted with caution.

The association between depressive symptoms and food craving has been studied primarily in clinical cohorts with mood or eating disorders, where positive associations have been observed (Muftuoglu et al., 2018). Studies in non-clinical cohorts are limited and inconsistent: One study using ecological momentary assessment found no association between depressive mood and food craving but assessed these variables as momentary states and used limited measures (Aulbach et al., 2025). A Japanese study found that experimentally induced depressive mood increased craving for Western food and rice, suggesting that depressive symptoms directly affect food craving, although this study assessed state rather than trait behavior (Fukuda, 2024). One study reported a particularly dysphoric mood before cravings in women, while others do not find a mood-craving link in women (Hill et al., 1991; Cohen et al., 1987). In addition, gender differences have been implied, with negative feelings linked to cravings in women and positive feelings in men (Lafay et al., 2001).

Despite inconsistent findings, psychophysiological models support the association between depressive symptoms and food craving, proposing mechanisms such as negative affect increasing the incentive value of food cues, resulting in cravings (Hepworth et al., 2010). Biological pathways involving brain serotonin, or classical and instrumental conditioning, may play a role (Hepworth et al., 2010). Indeed, induction of negative mood has been shown to increase appetite and attention to food cues, a key feature of craving (Hepworth et al., 2010). However, few studies have focused on *trait* food craving, limiting comparison. It is well documented that depressive symptom scores predict a higher BMI. A bidirectional relationship has been suggested, and longitudinal data indicate an effect of depressive symptoms on weight status (Luppino et al., 2010).

Mechanisms connecting depressive symptoms with trait eating behavior require further exploration. Nonetheless, speculations that depressive symptoms might impair executive function, such as inhibitory control, decision-making, and goal setting, and thereby affect trait eating behaviors, are supported by current research (Gibson, 2012). Additionally, depressive symptoms can be linked to blunted reward sensitivity with trait disinhibition, emotional eating, and food craving serving as a compensatory reward mechanism as they are directed toward “comfort food” high in fat and sugar (Gibson, 2012).

### 4.2 Association of trait eating behavior with BMI

The study found that higher trait food craving and trait disinhibition predicted a higher BMI, while cognitive restraint and restrained eating did not. Previous research has shown positive associations of trait disinhibition, and food craving with BMI (Muftuoglu et al., 2018; Bégin et al., 2012; Werrij et al., 2006; Wang et al., 2022). Although typically linked, trait emotional eating, uncontrolled eating, and hunger did not reach statistical significance after correction for multiple testing (Paans et al., 2018; Konttinen et al., 2010; Bégin et al., 2012; Löffler et al., 2015; Wang et al., 2022). Again, it must be acknowledged that the non-significant associations observed in the present analyses may be attributable to limited statistical power and should therefore be interpreted with caution. Previous conflicting findings, to which the present results contribute, suggest a more complex interplay of trait restraint and BMI. A U-shaped association and flexible versus rigid type of restraint have been proposed, adding to this complexity (Löffler et al., 2015).

### 4.3 Trait disinhibition mediates the association between depressive symptoms and BMI

Current findings indicate that trait disinhibition partially mediates the association between depressive symptoms and BMI. Although this has been suggested by numerous authors, studies on this mediation, especially in non-clinical groups, remain scarce. Previous research has often examined outcomes related to BMI rather than BMI itself; for instance, in prebariatric patients, trait disinhibition has been shown to mediate the association between depressive symptoms and binge eating (Cox and Brode, 2018). Other studies show that trait disinhibition connects depressive symptoms with increased savory and sweet food intake, implying that higher depressive symptoms boost energy intake through greater trait disinhibition (Konttinen et al., 2010). Additionally, trait disinhibition connects poor sleep quality with higher BMI, yet poor sleep alone cannot fully represent depressive mood, offering limited support for the present analyses (Blumfield et al., 2018). Although the present results demonstrate that trait disinhibition mediates the association between depressive symptoms and BMI, the magnitude of this effect is relatively small. Consequently, these findings should be interpreted with caution. In this context, it also has to be acknowledged that the proposed mechanisms remain theoretical, and causal inferences cannot be drawn from the present cross-sectional data. However, to date, no well-powered, large-scale studies appear to have reported published mediation effect sizes with trait disinhibition as a mediator of the association between depressive symptoms and BMI. Accordingly, the present study addresses this gap by providing initial empirical evidence for a theoretically proposed, but previously untested, pathway linking depressive symptoms, trait disinhibition, and BMI. Nonetheless, confirmatory longitudinal studies are needed.

Furthermore, *partial* mediation of trait disinhibition indicates additional factors linking depressive symptoms with BMI, such as a sedentary lifestyle, a dysregulated neuroendocrine response regulating hunger, or shared genetics and personality traits (Bégin et al., 2012; Werrij et al., 2006; Clum et al., 2014). However, in the present analyses, we did not find state hunger to be associated with depressive symptoms, BMI, or trait eating behaviors. The influence of specific personality traits, genetic factors, or lifestyle factors warrants exploration in future research.

Trait food craving was not identified as a mediator despite meeting the Baron and Kenny criteria for testing mediation. The association between depressive symptoms and BMI through increased food craving is often suggested but lacks evidence. This aligns with the present findings, which indicate associations between these variables but do not confirm mediation. The broader nature of assessed food craving encompasses various food types, not all of which may be equally relevant to weight gain or mood regulation. Craving for particularly carbohydrate-rich foods may be more relevant in the context of depressive symptoms and BMI. Studies indicate that individuals experiencing depressive symptoms may exhibit selective craving for carbohydrate-dense foods (Thurn et al., 2025). These targeted cravings, rather than general food craving tendencies, might drive caloric intake and subsequent weight gain in individuals with depressive symptoms. The lack of a mediating effect may reflect a mismatch between the specific mechanism, such as carbohydrate craving as a response to mood dysregulation, and the broad measure of craving. Future research investigating mediation should focus on specific food cravings, particularly carbohydrates.

Emotional eating has been recurrently reported as a mediator linking depressive symptoms with BMI, but testing this hypothesis was unjustified, as no association was found between emotional eating and BMI after adjustment for multiple testing (Clum et al., 2014; Lazarevich et al., 2016; van Strien et al., 2016). Given the exploratory nature of the present study, we adhered to strict premises.

### 4.4 Strengths and limitations

The study has strengths in addition to some limitations. The strengths are the exclusion of individuals with existing mood or eating disorders and antidepressant treatment, which can profoundly affect weight status and eating behavior, thereby enhancing the generalizability of results. Nonetheless, several limitations must be acknowledged: The cross-sectional study design precludes causal inference, a limitation that similarly applies to the interpretation of mediation models. Future longitudinal investigations are required to establish temporal precedence and elucidate the underlying causal mechanisms. Furthermore, the generalizability of the findings is limited by the single-center recruitment strategy. In addition, the generalizability of the findings may be limited by self-selection bias, as the study title (“Obese Taste Bud”) may have preferentially attracted individuals with particular food- or eating-related interests. Moreover, the exclusive use of self-report instruments may introduce systematic bias arising from social desirability tendencies and awareness bias. Furthermore, the underlying mechanism remains speculative as we did not assess actual eating behavior or brain imaging in this cohort. The relatively small sample size (*N* = 77) likely constrained the statistical power of the analyses, thereby contributing to the loss of significance after correction for multiple comparisons. Nevertheless, a highly stringent statistical workflow enabled critical and meaningful data analysis. Future studies should corroborate the present findings in larger, longitudinal cohorts. We did not perform separate analyses for women and men, as statistical power was limited. However, the present results do not suggest meaningful gender differences in this cohort.

## 5 Conclusion

These findings suggest that depressive symptoms, even in a spectrum where depressive mood does not meet the diagnostic criteria of depressive disorders, can meaningfully influence stable patterns of eating behaviors and weight status. Importantly, we identified trait disinhibition as a significant partial mediator linking depressive symptoms and BMI. This highlights a potential behavioral mechanism through which depressive symptoms may contribute to the current obesity pandemic and provides targets for future interventions.

## Data Availability

The raw data supporting the conclusions of this article will be made available by the authors, without undue reservation.
